# Factors associated with unsafe sexual behavior among sexually active Chinese University students, Hebei Province, 2019

**DOI:** 10.1186/s12889-021-11992-2

**Published:** 2021-10-21

**Authors:** Zhengjia Ren, Yuchu Zhou, Yanhong Liu

**Affiliations:** 1 Present Address: Department of Clinical Psychology, The Third Affiliated Hospital of Chongqing Medical University, Chongqing, China; 2 Present Address: Daka Education Consulting Co. Ltd, Chengdu, Sichuan China; 3 Department of Psychology, Hebei University of Chinese Medicine, Wuhan, Hebei China

**Keywords:** HIV knowledge, Unhealthy sexual behavior, Multiple sexual partners, Online dating, Condom use, University students

## Abstract

**Background:**

Healthy sexual behavior is critical for controlling the HIV/AIDS epidemic among university students, particularly in regions with increasing infection rates among university students in China.

**Methods:**

This study investigated the prevalence of unhealthy sexual behavior in the past 6 months and the associated demographic and psychosocial factors among sexually active Chinese university students. Self-identified sexually active Chinese university students were recruited for the study.

**Results:**

The study found that most students used condoms inconsistently during sexual intercourse (54.8%), and logistic regression showed that condomless sex was associated with being raised by a single parent (AOR = 1.934, 95% CI 1.234–3.031) or by grandparents or others (AOR = 1.583, 95% CI 1.003–2.50) and with sometimes using dating apps (AOR = 1.496, 95% CI 1.106–2.024). The independent protective factors for condomless sex were HIV knowledge scores between 15 and 18 compared to scores of 0 to 4 (AOR = 0.434, 95% CI 0.244–0.771). Among sexually active university students, 15.5% reported that they had multiple sexual partners; having multiple sexual partners was associated with sometimes (AOR = 2.543, 95% CI 1.553–4.167) or always (AOR =4.048, 95% CI 2.177–7.527) using dating apps. Being female (AOR = 0.402, 95% CI 0.231–0.699) and in a relationship (AOR = 0.236, 95% CI 0.154–0.363) were protective factors against having multiple sexual partners.

**Conclusions:**

There is an urgent need to promote HIV prevention and to implement psychosocial interventions by providing comprehensive sex education and access to condoms and health care on campuses to decrease the potential factors related to unhealthy sexual behaviors among university students.

## Introduction

HIV/AIDS prevalence among Chinese university students has been drastically increasing through sexual transmission, with an annual growth rate ranging from 30 to 50% in the last five years [1]. Engaging in condomless sex (59%) and having multiple sexual partners (66.7%) are very common types of sexual behavior among sexually active university students, and these behaviors usually put them at increased risk for HIV infection [2].

The alarmingly high (3000 new cases every year) and still increasing HIV/AIDS annual growth rate (30–50%) among Chinese university students is mainly related to sexual practices, which suggests that the foundation of HIV infection prevention and intervention is an understanding of the factors related to the sexual practices of university students [1]. A research survey (2003) of 22,493 unmarried Chinese undergraduate university students revealed that 17.6% of males and 8.6% of females reported being sexually active, and more than one-third of sexually active students had engaged in condomless sex [3]. Yan (2006) et al. found that 18.1% of 4769 Chinese female undergraduates were sexually active and that 29.32% of these sexually active female students had had multiple sexual partners in their lifetime [4]. Various studies show that in contrast to monogamous relationships, having multiple sex partners is associated with the clustering of sexual risk behaviors and their synergistic promotion of HIV transmission [5, 6]. People with large numbers of sex partners are more vulnerable to sexually transmitted diseases, and sexually transmitted disease transmission is particularly heightened by partner change [7]. Previous scholars have claimed that partner reduction is related to declines in HIV incidence, and sexual behavior changes are a crucial approach to HIV prevention [8]. Understanding the factors associated with these two unhealthy sexual behaviors is pivotal. An increasing number of researchers argue that contextual factors are associated with sexual behavior.

Generally, adolescents spend most of their time at school or in a family environment, and the quality of their environment plays a foundational role in individuals’ health behaviors [9]. Scientific evidence indicates that certain factors from the family, school and social context protect against sexual risk behavior. Previous research has indicated that living with parents and experiencing greater parental involvement and supervision have positive impacts leading to fewer sexual partners and condom use [10–12]. At school, a greater commitment to schoolwork and programs about sexual education are associated with later sexual initiation, fewer sexual partners, and the use of protection, such as condoms or contraceptives [13, 14]. Previous researchers have discovered that poor knowledge of HIV infection is common and is correlated with high-risk sexual behavior, which is a challenge for HIV/AIDS control in China [15, 16].

The social context also plays a role in the development of risk behaviors. Changes in dating and dating styles are also accompanied by changes in the social environment. Internet dating has gained popularity among Chinese university students as a means to socialize and find potential sexual partners. The increasing use of dating apps has created new potential risk environments because people can easily engage in casual sex, which may facilitate having multiple sexual partners or unsafe sex. Previous research has suggested that the increased use of dating apps is associated with unsafe sexual behaviors, such as having condomless sex and multiple sexual partners [17, 18].

The issue of unsafe sexual practices among university students is complicated and involves different dimensions, such as family, school, and social context [12, 19]. An important issue in identifying points of leverage for improving students’ safe sexual practices is determining factors affecting safe sexual practices. Such knowledge might inform parenting practices as well as school-based sexual education policies, practices, and interventions. In the era of an alarming HIV/AIDS epidemic (3000 new cases annually) among university students, it is crucial to understand the determinants of unhealthy sexual practices among university students to inform future policies and programs that help protect them. The objective of this study was to investigate factors associated with unhealthy sexual behavior in the past 6 months among sexually active Chinese university students.

## Methods

### Procedure

An anonymous online survey was created using Wenjuanxing, a Chinese online questionnaire collection platform. Survey links were distributed by the project coordinator at four universities by using convenience sampling from September to December 2019. The researchers posted a description of the study objectives and benefits, a confidentiality guarantee, and the consent form online. Briefly, participants were recruited from the four public universities in Hebei Province, which cover a wide range of disciplines, such as science, engineering, business, sports, and medicine.

### Participants

The present study is part of a larger project on HIV knowledge among university students. A total of 6700 students participated in the larger project, and 901 (13.4%) of these participants who self-identified as sexually active Chinese university students were identified in the present study. The 901 participants ranged in age from 16 to 26. Our inclusion criterion was that the participants responded to the question on history of sexual intercourse and reported a history of sexual intercourse (vaginal intercourse or anal intercourse) in the past half year, which was the definition of sexually active we used in our study. In the questionnaires, gender (male or female), age, grade (freshman, sophomore, or junior and above), area of origin (city: it refers to a large residential area formed by agglomeration of non-agricultural industries and non-agricultural population; town: it refers to certain scale of industrial and commercial residential areas, where more than 50% of the population is not agricultural population, and it is usually smaller than a city and larger than a village; village: it refers to a small community or group of houses in a rural area, where the population is more scattered and whose main source of livelihood is agriculture), university attended (science and engineering, business, sports, medicine), relationship status (single or in a relationship), and caregivers in childhood were assessed. One item asked who the respondent’s caregiver was in childhood, with the following response options: 1. parents, 2. father or mother, and 3. grandparents or others.

#### Frequency of use of online dating apps

One item measured participants’ frequency of use of online dating apps and was included in the analysis. Participants were asked to rate how often they used the internet to meet or socialize for the purpose of dating, including on social network websites and mobile social applications (0 = *never*, 1 = *sometimes*, 2 = *usually*).

### Unsafe sexual behavior

Condomless sex**.** One item asked whether (yes or no) the respondent had sexual intercourse without a condom: (1) Have you had sexual intercourse in the past half year without a condom?

#### Number of sexual partners

One question asked about the student’s number of sexual partners in the past 6 months. If the student had had sexual intercourse with more than one person (two or above), we coded their response as ‘multiple sexual partners’.

#### HIV knowledge

HIV knowledge was measured with the *Brief HIV Knowledge.*

*Questionnaire* (HIV-KQ-18) [20]. The HIV-KQ-18 has been demonstrated to have good internal consistency and construct validity in research with college students, men who have sex with men, and various populations in China [21, 22]. The instrument has a total of 18 questions that are scored by assigning “1” to questions correctly answered and “0” to questions incorrectly answered.

### Statistical analysis

Descriptive statistics were calculated. To assess the relative contribution of each of the predictor variables, a logistic regression analysis was carried out. All the variables were entered into the model using a block entry approach. Adjusted odds ratios (AORs) and 95% confidence intervals (CIs) were calculated for each predictor variable. All *P*-values < 0.05 were considered statistically significant. The Statistical Package for the Social Sciences (SPSS) version 19.0 was used for data analysis. The difference in the total HIV knowledge scores between sexually active students and non-sexually active students was compared by an independent t-test. The differences in the correct answer rate for each question on the HIV-KQ 18 between sexually active students and non-sexually active students was determined using chi square (χ2) tests.

### Ethical issues

Participants were informed that the study was voluntary and anonymous and that they could withdraw from the study at any time. The study was approved by the Ethics Committee at Hebei University of Chinese Medicine.

## Results

### Sample description

Table 1 presents the sociodemographic information of the 901 sexually active university students. All 901 respondents reported being sexually active in the past half year. Of the students, 65.8% were male and 34.2% were female. Other detailed demographic data are summarized in Table 1.

**Table 1:** Demographics, STI-related sexual behaviors, and HIV knowledge among sexually active Chinese university students, Hebei Province, 2019

Participants		*N* = 901(%)
**Sex**	Male	593 (65.8)
	Female	308 (34.2)
**Age**	16–20	638 (70.8)
	21–26	263 (29.2)
**Education**	Freshman	344 (38.2)
	Sophomore	303 (33.6)
	Junior and above	254 (28.2)
**Area where the student grew up**	City	251 (27.9)
	Town	215 (23.9)
	Village	435 (48.3)
**University**	Science and engineering	135 (15.0)
	Business	34 (3.8)
	Sports	471 (52.2)
	Medical	261 (29.0)
**Relationship status**	Single	158 (17.5)
	In a relationship	743 (82.5)
**Caregiver in childhood**	Parents	702 (77.9)
	Father or mother	104 (11.5)
	Grandparent or others	95 (10.5)
**Frequency of use of dating apps**	Never	408 (45.3)
	Sometimes	372 (41.3)
	Always	121 (13.4)
**Condomless sex in the past 6 months**	Yes	407 (45.2)
	No	494 (54.8)
**Multiple sexual partners in the past 6 months**	Yes	140 (15.5)
	No	761 (84.5)
**HIV knowledge**	0–4	96 (10.7)
	5–9	202 (22.4)
	10–14	431 (47.8)
	15–18	172 (19.1)

#### Sexual behaviors

Of the 901 respondents, 15.5% had two or more sexual partners in the past half year, and among those who had more than one sexual partner, 74.3% (106/140) used condoms inconsistently. A total of 54.8% (494/901) of participants reported using condoms inconsistently; among those participants who used condoms inconsistently, 21.4% (106/494) had multiple sexual partners. A total of 11.8% of the 901 participants reported these two behaviors in the past half year.

### HIV knowledge

Of all the sexually active students, 10.7% had scores between 0 and 4, 22.4% had scores between 5 and 9, 47.8% had scores between 10 and 14, and 19.1% had scores between 15 and 18. The Cronbach’s α of the HIV-KQ-18 in this sample was 0.83. Independent sample t-tests were used to determine the difference in HIV knowledge between sexually active students and non-sexually active students. The results revealed a significant difference in HIV knowledge between sexually active students and the other students, t (6698) = − 2.99, *p* = 0.003. This finding suggests that sexually active students (10.71 ± 4.20) had significantly lower HIV knowledge scores than non-sexually active students (11.15 ± 4.15). Our study found that for the vast majority of questions, the sexually active group (Q1, 2, 6, 7, 8, 9, 13, 15, 16) had significantly fewer correct answers than the non-sexually active group. Sexually active students received higher scores on questions 3, 12, 17, and 19 than non-sexually active students, but the correct answer rate was still low. Details of the percentages of sexually active students who answered each question correctly are shown in Table 2.

**Table 2:** Percentages of correct answers to HIV knowledge items (HIV-KQ-18) among sexually active students (SAS) and non-sexually active (NSAS) students

	SAS N = 901(%)	NONSAS ***N*** = 5799(%) N = 5799	X2/p
1. Coughing and sneezing DO NOT spread HIV.	69.3	77.8	< 0.001
2. A person can get HIV by sharing a glass of water with someone who has HIV.	55.6	67.7	< 0.001
3. Pulling out the penis before a man climaxes/cums keeps a woman from getting HIV during sex.	81.2	63.8	< 0.001
4. A woman can get HIV if she has anal sex with a man.	78.5	77.4	0.49
5. Showering or washing one’s genitals/private parts after sex keeps a person from getting HIV.	73.7	72.2	0.61
6. All pregnant women infected with HIV will have babies born with AIDS.	40.4	56.0	< 0.001
7. People who have been infected with HIV quickly show serious signs of being infected.	75.9	85.2	< 0.001
8. There is a vaccine that can stop adults from getting HIV.	55.9	65.8	< 0.001
9. People are likely to get HIV by deep kissing, putting their tongue in their partner’s mouth, if their partner has HIV.	45.6	53.5	< 0.001
10. A woman cannot get HIV if she has sex during her period.	75.7	76.1	0.81
11. There is a female condom that can help decrease a woman’s chance of getting HIV.	62.6	63.1	0.75
12. A natural skin condom works better against HIV than does a latex condom.	29.5	22.3	< 0.001
13. A person will NOT get HIV if she or he is taking antibiotics.	57.2	61.3	0.02
14. Having sex with more than one partner can increase a person’s chance of being infected with HIV.	88.0	87.5	0.69
15. Taking a test for HIV one week after having sex will tell a person if she or he has HIV.	29.4	37.7	< 0.001
16. A person can get HIV by sitting in a hot tub or a swimming pool with a person who has HIV.	52.4	63.8	< 0.001
17. A person can get HIV from oral sex.	45.9	38.9	< 0.001
18. Using Vaseline or baby oil with condoms lowers the chance of getting HIV.	55.0	45	< 0.001

#### Frequency of use of online dating apps

Of all respondents, 45.3% reported that they never used dating apps, 41.3% reported sometimes using dating apps, and 13.4% reported always using dating apps.

### **Logistic regression analysis**–**factors associated with** condomless **sex**

In the logistic regression analysis, the independent risk factors for condomless sex were being raised by either one’s mother or one’s father (AOR = 1.934, 95% CI 1.234–3.031) or by grandparents or others (AOR = 1.583, 95% CI 1.003–2.50) compared to being raised by both parents as well as sometimes using dating apps (AOR = 1.496, 95% CI 1.106–2.024) compared to never using dating apps. The independent protective factors for condomless sex were having an HIV knowledge score between 15 and 18 compared to a score between 0 and 4 (AOR = 0.434, 95% CI 0.244–0.771). The results are shown in Table 3.

**Table 3:** Logistic regression of demographics and HIV knowledge on multiple sex partners and condom inconsistency in the past 6 months among sexually active Chinese university students, Hebei Province, 2019

	Variables	Multiple sexual partners			Condom inconsistency		
		P	AOR	95%C.I.	P	AOR	95%C.I.
**Sex**	Male	Referent			Referent		
	Female	.001	.402	.231–.699	.177	.805	.588–1.103
**Age**	16–20	Referent			Referent		
	21–26	.465	.794	.428–1.474	.434	.853	.574–1.269
**Education**	Freshman	Referent			Referent		
	Sophomore	.023	.564	.345–.924	.932	.985	.706–1.375
	Junior and above	.280	.687	.348–1.358	.154	1.389	.884–2.183
**Area where the student grew up**	City	Referent			Referent		
	Town	.282	.738	.425–1.283	.870	1.032	.706–1.509
	Village	.080	.655	.407–1.052	.357	1.168	.840–1.624
**University**	Science and engineering	Referent			Referent		
	Business	.424	.521	.106–2.575	.236	.618	.279–1.371
	Sports	.071	1.805	.950–3.430	.705	1.087	.707–1.670
	Medical	.764	1.124	.526–2.402	.331	1.253	.795–1.973
**Relationship status**	Single	Referent			Referent		
	In a relationship	.000	.236	.154–.363	.203	.788	.545–1.137
**Caregiver in childhood**	Parents	Referent			Referent		
	Father or mother	.394	.738	.367–1.485	.004	1.934	1.234–3.031
	Grandparent or others	.084	1.662	.934–2.958	.049	1.583	1.003–2.500
**Frequency of use of dating apps**	Never	Referent			Referent		
	Sometimes	.000	2.543	1.553–4.167	.009	1.496	1.106–2.024
	Always	.000	4.048	2.177–7.527	.114	1.414	.920–2.174
**HIV knowledge**	0–4	Referent				Referent	
	5–9	.870	.945	.480–1.861	.774	.928	.555–1.551
	10–14	.955	.981	.515–1.872	.286	.767	.470–1.249
	15–18	.284	.622	.261–1.482	.004	.434	.244–.771

### Logistic regression analysis–factors associated with having multiple sexual partners

The logistic regression analysis showed that independent risk factors for having multiple sexual partners were sometimes (AOR = 2.543, 95% CI 1.553–4.167) or always (AOR =4.048, 95% CI 2.177–7.527) using dating apps compared to never using dating apps. The independent protective factors for having multiple sexual partners were being female (AOR = 0.402, 95% CI 0.231–0.699) and in a relationship (AOR = 0.236, 95% CI 0.154–0.363).

## Discussion

In this study, both sexually active and non-sexually active students were deficient in HIV knowledge, but the sexually active students were more deficient. We found that for sexually active students, increased knowledge was associated with a lower likelihood of using condoms inconsistently. This is consistent with previous studies showing that university students without accurate and comprehensive HIV knowledge are less likely to adopt healthy sexual practices [23, 24]. Additionally, the present study found that higher-frequency users of dating apps reported having multiple sex partners and using condoms inconsistency. Poor knowledge of HIV and the fact that sexually active college students are more likely to find potential sexual partners through dating apps make college students more likely to engage in risky sexual behavior. Our finding of the poor HIV knowledge and high likelihood of dating app use among sexually active students emphasizes the need for concerted effort toward educating Chinese university students about HIV.

The current study also revealed that university students who were raised by a single parent or grandparents were less likely to use condoms consistently during sexual intercourse than individuals who grew up with both their parents. A previous researcher noted that adolescents who grow up in single-parent households are less likely to have control over their sexual behaviors than adolescents raised by both parents due to less parental supervision and lack of communication of sexual knowledge; these issues may contribute to a lack of sexual knowledge among teenagers and may lead to unhealthy sexual behaviors, suggesting the importance of family influences on sexual behaviors [25]. The current study implies that compared to those raised by both parents, individuals who grow up with grandparents or a single parent may have disadvantages in terms of their understanding of social norms, access to economic resources, access to healthcare information, and social influences. Future services need to focus more on these populations, providing them with more information, social support, social connections, HIV information, easy access to testing, and medical services.

Consistent with a previous study, the current study revealed an alarming lack of HIV/AIDS knowledge and prevention among university students, and a low level of HIV knowledge was correlated with condomless sex [26]. Researchers have reported that knowledge about HIV/AIDS, as a form of self-empowerment, may influence a person’s perception of risk, which can lead to behavior change [27, 28]. The current work suggests that providing comprehensive HIV/AIDS education in universities and even before college is critical. Schools and families need to design appropriate sex education for individuals at different stages of development to teach them how to maintain and develop healthy intimate relationships. At universities, compulsory sexual health education courses should be offered that incorporate the latest knowledge about safe sex and HIV/AIDS prevention into the syllabus, including the proper use of condoms. Such courses should empower students to avoid unhealthy sexual behaviors and sexually transmitted diseases. Additionally, posters and social media advertisements about safe sex and HIV/AIDS prevention knowledge should be posted around campus and on campus social media to make knowledge about sexual health more widespread. There is also a need to reduce the stigma of sexually transmitted diseases by providing easy access to condoms and testing at universities in a way that protects students’ privacy. The study found that single people were more likely to have multiple sexual partners, possibly reflecting that single college students who are currently sexually active may tend to engage more in casual sex. As a result of having multiple sex partners and greater odds of not using protection against sexually transmitted diseases, these individuals have increased opportunity to contract such diseases. Our results support continued efforts to tailor certain interventions to sexually active university students who have casual sex.

Dating apps have become popular social networking tools for the purpose of dating and seeking sexual partners; they enable individuals to connect with other potential dating partners efficiently at any time and any place, allowing them to choose to engage in casual sex more easily than before. A previous study claimed that the increasing number of users of dating apps makes it easier for gay men and bisexual individuals to choose to engage with multiple sexual partners and in unsafe sex [29, 30]. The present study expands on previous research, showing that dating app use is associated with multiple sexual partners among college students. Therefore, health promotion workers and designers of dating apps should incorporate preventive messages and notifications into the apps to promote safe sex practices. In addition, school-based interventions should empower students to negotiate safe sex via dating apps, with a focus on condom use, consent for sex behaviors, sexual positioning, testing status, and obtaining information on the health status of sexual partners, etc. Our findings support previous work that has shown that those with multiple partners are most likely to be young men, which implies that sexual health promotion and education should pay more attention to this group [31, 32]. Gender differences in having multiple partners may reflect that cultural values can affect sexual behavior. Traditional values that proscribe extramarital sex and enforce virginity for women are prevalent in Chinese culture and may have a strong influence on Chinese women’s behavior.

### Limitations

This study also had some limitations. Foremost, the current study evaluated only two sexual behaviors in students from four universities in one province of China, and the cross-sectional nature of the current study prevented us from drawing conclusions about causal effects. Any generalization of the results should be performed with caution. Second, the sensitive nature of sexual behaviors such as sexual practices, the number of sexual partners, and condom use might have led to the underreporting of these behaviors because of social desirability bias. Third, we did not ask about the stability of or changes in the respondents’ relationships in the past 6 months. Therefore, the current study did not confirm whether the students who reported using condoms inconsistently were in a monogamous relationship, which may not put someone at risk for HIV or sexually transmitted diseases. In addition, the study asked only about sexual behavior in the last six months. Last, several variables related to sexual behaviors (drug/alcohol use) were not evaluated, and the relevant conclusions should be interpreted with caution.

## Conclusion

This study investigated the prevalence of and factors associated with unhealthy sexual behaviors related to HIV/AIDS transmission among university students. First, the current findings highlight that developing relevant strategies to address poor knowledge of HIV/AIDS is key to improving safe sexual practices. Second, tailored interventions that help students negotiate safe sex and healthy relationships via dating apps should be implemented.

### Suggestions for future directions

To reduce the spread of HIV/AIDS and other sexually transmitted diseases on college campuses, it is very important to understand the risk factors affecting unsafe sexual behaviors among college students and make timely interventions to tackle these risk factors. In the future, it is necessary to offer sexual health education courses that are designed to engage students and to evaluate students’ understanding of knowledge related to HIV/AIDS prevention and treatment in order to empower students to take actions to protect themselves and increase their preventive behaviors. HIV/AIDS prevention at universities needs to focus on high-risk groups, and self-protection strategies need to be promulgated on college campuses where dating apps are used to make friends.

## Data Availability

The data that support the findings of this study are available from the corresponding author upon reasonable request.

## Abbreviations

HIV-KQ-18: Brief HIV Knowledge Questionnaire
AIDS: Acquired immunodeficiency syndrome
HIV: Human immunodeficiency virus (HIV)
LGBT: Lesbian, gay, bisexual, and transgender
STDs: Sexually transmitted diseases
